# Increased Musashi-2 and Decreased NUMB Protein Levels Observed in Human Colorectal Cancer are reverted to Normal Levels by ATRA-Induced Cell Differentiation

**DOI:** 10.33140/ijcrt/03/02/00003

**Published:** 2018-08-15

**Authors:** Lynn M Opdenaker, Ryan Kowash, Gabriel Masters, Bruce M Boman, Tao Zhang, Shirin R Modarai

**Affiliations:** 1 Center for Translational Cancer Research, Helen F. Graham Cancer Center & Research Institute, Newark, DE; 2 Dickinson College, Carlisle, PA; 3 Hamilton College, Clinton, NY; 4 University of Delaware, Newark, DE; 5 Childrens Hospital of Pennsylvania, Philadelphia PA

**Keywords:** Musashi-1, Musashi-2, NUMB, Colorectal Cancer, Differentiation

## Abstract

**Background:**

Musashi stem cell (SC) proteins (MSI-1 & MSI-2) are known to become over expressed during colorectal tumorigenesis in humans and mice. MSI-1 overexpression induces tumorigenesis through Notch activation via inactivation of NUMB. Previous studies also show that MSI-2 overexpression in mice induces intestinal tumorigenesis but the mechanism is independent of NUMB. However, whether the MSI-2/NUMB pathway contributes to colorectal cancer (CRC) development in humans is still undetermined.

**Methods:**

We evaluated expression of MSI-2 and NUMB proteins in matched normal and CRC patient samples, as well as in human CRC cell lines. We also determined whether induction of cellular differentiation by all-trans retinoic acid (ATRA) influences MSI-2 and NUMB expression.

**Results:**

Analysis of matched patient tissue samples and CRC cell lines showed that MSI-2 protein expression is significantly increased and NUMB expression is decreased in CRCs compared to the normal colonic tissue. Immunostaining of normal and adenomatous colonic epithelium revealed that MSI-1+ andMSI-2+ SCs reside in the SC niche and they become overpopulated during colon tumorigenesis. Moreover, promoting cellular differentiation by ATRA reduces MSI-2 protein levels, while increasing NUMB protein levels in human CRC cell lines.

**Conclusions:**

MSI-2/NUMB protein expression is altered during colon tumorigenesis, and indicates that MSI-2/NUMB signaling in human colonic stem cells is closely linked to normal colonic epithelial homeostasis.

**Implications:**

The ability to normalize MSI-2/NUMB signaling by inducing differentiation of cancer SCs suggests a novel therapeutic approach for CRC treatment.

## Introduction

A substantial body of research on the molecular etiology of colorectal cancer (CRC) reveals that overpopulation of stem cells (SCs) drives tumor initiation and progression, but the underlying mechanisms that regulate SCs and that become dysregulated in CRC development are not well understood [1–4]. Musashi proteins (MSI-1 & MSI-2) have been found to be reliable markers for normal and malignant colonic SCs and both proteins become overexpressed during colon tumorigenesis in humans and mice [5–7]. Because SCs play an important role in maintaining normal colonic homeostasis and dysregulation of the SC population leads to colon tumorigenesis, identification of key proteins and pathways associated with colonic SC dysregulation is crucial.

In the colon, regulation of gene expression in SCs is a key determinant in controlling cell-fate specification, but the regulation at the translational level is incompletely understood [8]. MSI-1 and MSI-2 are RNA binding proteins that bind and regulate the stability of mRNAs and subsequent translation, particularly those associated with oncogenic signaling pathways such as NUMB/NOTCH, Wnt, and P21 [9]. Additionally, MSI-1 and MSI-2 expression is integral to maintaining the balance between self-renewal and differentiation in the SC population [10].

In normal SCs, MSI-1 is highly expressed, while differentiating cells exhibit decreased levels of expression. In addition, expression of MSI-1 is elevated in cancer SCs and solid tumors [10]. In model organisms, such as *C. elegans*, MSI-1 has been shown to positively regulate Notch signaling through the inactivation of NUMB (a NOTCH signaling repressor) [8]. Additionally, MSI-1 has been identified as an important oncoprotein in CRC and most recently as a regulator of CRC growth [11–13]. Notably, MSI-1 expressing cells reside in the bottom of human colonic crypts mostly within the SC niche in normal colonic epithelium where ALDH+ SCs also reside [5,14]. Moreover, MSI-1 appears to play a role in modulating crypt cell homeostasis within the crypt base [15].

On the other hand, information on MSI-2 is just beginning to emerge. In addition to elevated levels of MSI-1, various tumors also express higher levels of MSI-2 and both proteins are linked to inflammation in CRC [16]. Recent research on MSI-2 in mice has also shown that overexpression of MSI-2 contributes to transformation of intestinal epithelium and this is due to MSI-2’s ability to promote crypt fission and expand the intestinal SC population [7]. However, the mechanism for MSI-2 induction of tumors in mice was independent of NUMB. Whether the MSI-2/NUMB pathway contributes to CRC development in humans is still undetermined.

The objective of this study is to analyze the expression of MSI-1 and MSI-2 in matched normal and tumor patient samples, as well as in human CRC cell lines. Our study was also designed to determine whether induction of cellular differentiation by all-trans retinoic acid (ATRA) influences MSI-2 and NUMB expression. Our main goal was to gain insight into the role of MSI-2 in human CRC development.

## Material and methods

### All-trans Retinoic Acid (ATRA) treatment

Cells were plated at 100,000 cells per well of a 6-well tissue culture plate. Cells were plated and allowed to attach for 24 hours and then serum starved for 24 hours prior to drug treatment. HT29 cells were treated at the IC50 value of ATRA (1μΜ) for 2 days and SW480 cells were treated at the IC50 value of ATRA (10μΜ) for four days before western blot analysis of protein expression. DMSO was the vehicle control used for all ATRA treatments. IC50 values were previously published [17].

### CRC cell culture

HT29 and SW480 colorectal cancer cell lines were purchased from ATCC and were grown in McCoys medium (HT29) and L-15 medium (SW480). All cell culture media contained 10% FBS and 1% penicillin/streptomycin. The cells were grown in 5% CO_2_ and 95% air at 37°C.

### Human patient samples

All research involving human colonic tissue was performed in accordance with the Declaration of Helsinki and was approved by the appropriate Institutional Review Board (FWA0000655) at Christiana Care Health Services, Inc (Newark, DE). Tissue samples were either obtained or processed on the day of surgery or archived previously consented tissue samples were used from the hospital.

### Immunofluorescence

Colon tissue samples were obtained from patients, paraffin embedded, and microscope slides were prepared with colon tissue sections that were less 10μm in size. The slides were washed and hydrated using CitraSolv and ethanol before heating the slides submerged in Antigen Retrieval Solution (lx Citra). After washing again with Phosphate-buffered saline (PBS), the sections were blocked overnight at 4°C with a goat serum-derived blocking buffer. The next day the tissue sections were incubated with primary antibody (MSI-1 1:100 and MSI-2 1:100) and left overnight at 4°C. The slides were washed in lx PBS and then the Alexa Fluor 488 conjugated secondary antibody (1:200) was added at 37°C for one hour. The samples were washed again in 1x PBS before staining the slides with a 1:10,000 dilution of Hoescht stain for ten minutes. The slides were then cleaned and a mounting medium was applied before placing a coverslip on the slide and sealing the edges. The slides were then viewed and images recorded on the Zeiss Fluorescent inverted microscope.

### Immunohistochemistry (IHC) staining

IHC was performed as we previously described using rabbit polyclonal anti-Musashi antibody (1:200, Bioss Antibodies, Woburn MA) that reacts against both human MSI-1 and MSI-2 proteins [14].

### Western blotting

Total cellular protein was collected using RIPA Lysis Buffer containing a protease inhibitor cocktail. Protein concentrations were determined using a BCA assay (Pierce). The samples were heated at 95 °C for 10 minutes and then were subjected to SDS-PAGE on a 12% polyacrylamide gel for 90 minutes at 100V. The gel was transferred to an activated PVDF membrane for 1hour at 100V. The blot was placed in 3% BSA and blocked overnight on a shaker at 4°C. All primary antibody incubations were performed overnight on a shaker at 4°C (MSI-1 1:1,000, MSI-2 1:2,000, NUMB 1:1,000, c-MYC 1:1,000, beta actin 1:10,000) and secondary antibody incubations were one hour at room temperatures. All primary antibodies were purchased from Abeam. The protein bands were visualized via chemiluminescence using an ECL kit (Pierce) and detected on the G: box Chemi Imager (Syngene).

### Statistical analysis

The data was expressed as mean ± SEM (n=3). A student t-test was performed to compare results between normal and tumor patient samples and control and treated samples.

## Results & discussion

### Expression of MSI-1, MSI-2, NUMB, and C-MYC in matched patient samples

**Figure 1:**
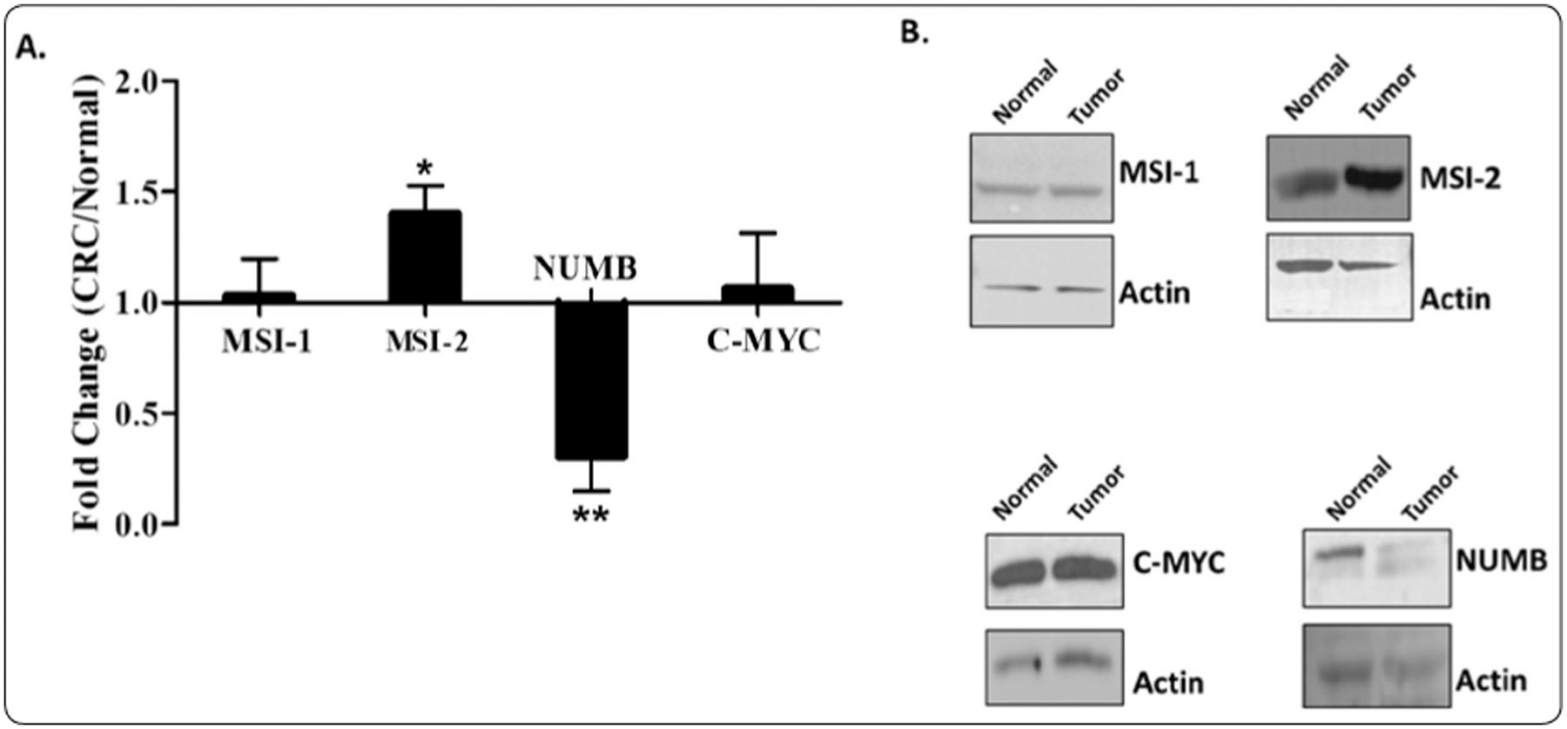
Expression of MSI-1, MSI-2 and downstream Musashi targets NUMB and C-MYC in matched normal and tumor patient samples. Matched patient samples were from the normal colonic epithelium and CRC tissue of the same patient. (A) The graph represents fold change of CRC over normal colonic tissue with ± SEM. Results show that MSI-2 has elevated protein expression and NUMB has reduced protein expression in CRCs compared to normal colonic epithelium. Protein expression was quantified by densitometric analysis of western blots and normalized to beta actin. All samples were analyzed at N = 3–20. (B) Representative western blots of matched normal and tumor patient tissue lysates are shown for MSI-1, MSI-2, NUMB and C-MYC, as well as each corresponding beta actin.

Because it is still undetermined whether the MSI-2/NUMB pathway contributes to CRC development in humans, we evaluated the expression of MSI-2 and NUMB proteins in matched normal and CRC patient samples. Our western blot analysis of MSI-1 and MSI-2 protein expression showed that MSI-2 is significantly overexpressed in CRC tissues compared to matched normal tissues and that MSI-1 expression is only slightly increased (Figure 1).

We also analyzed NUMB and C-MYC proteins because they are downstream targets of the Musashi family of proteins. Muasahi represses the translation of NUMB, which activates Notch signaling. Our analysis showed that NUMB was significantly downregulated in CRC tissues as compared to matched normal tissues (Figure 1). The expression of C-MYC protein, which is a WNT target gene, did not change (Figure 1).

Our results have relevance to the role of MSI-2 in human colon tumorigenesis because several studies have previously investigated MSI-1 and reported that MSI-1 is expressed in low levels in normal colonic tissue and high levels in CRCs [18,19]. While less is known about MSI-2, a study by Wang, et al. reported that MSI-2 is overexpressed in human CRCs. This study also reported that MSI-2 becomes overexpressed in intestinal tumors in mice, but the mechanism is independent of NUMB. This finding on MSI-2 in mice differs from our results on humans showing that MSI-2 levels are high and NUMB levels are low in CRCs. However, our results indicate that MSI-2, like MSI-1, represses NUMB expression in CRC. It is known that MSI-1 binds to the 3’ untranslated region of NUMB, thus repressing its translation [7,20]. Repression of NUMB translation has important effects on NOTCH signaling. NUMB is a negative regulator of NOTCH signaling, inducing endosome-mediated degradation of NOTCH, thus preventing entrance to the nucleus and subsequent signaling [12]. Studies have found that MSI-1 upregulates NOTCH and Wnt while repressing the translation of NUMB [20,21]. Another study indicates that MSI-1 plays a role in maintaining SCs through the Notch signaling pathway in other mammalian cells [20]. Forced expression of activated NOTCH increased Musashi levels, whereas silencing NOTCH via short hairpin RNA reduces Musashi levels in CRC cells [12].

MSI-2 has also been found to inhibit NUMB expression in other tissues, which concurs with our findings on MSI-2 and NUMB in CRC [22]. We found that MSI-2 levels are increased to a greater extent than MSI-1. To date, little research has been completed on how MSI-1 and MSI-2, in tandem, affect NUMB and eventually Notch signaling.

Although we did not see that C-MYC expression is substantially altered in our CRC samples, other studies have reported C-MYC expression is elevated in many human CRCs but that there is a wide range of C-MYC protein expression [23,24]. Notably, C-MYC, like NUMB, is a downstream target of the Musashi family of proteins. In addition, C-MYC is a known WNT target gene. WNT signaling is activated in multiple cancer types, including CRC and activation of the WNT signaling pathway up-regulates C-MYC expression and promotes colon tumorigenesis [25]. Nonetheless, while our findings show the expression of MSI-2 and NUMB is significantly altered between the normal and CRC tissues, we did not find that the expression of C-MYC to be substantially different between matched patient tissues.

### The expression of MSI-1, MSI-2, and NUMB in CRC cell lines

**Figure 2:**
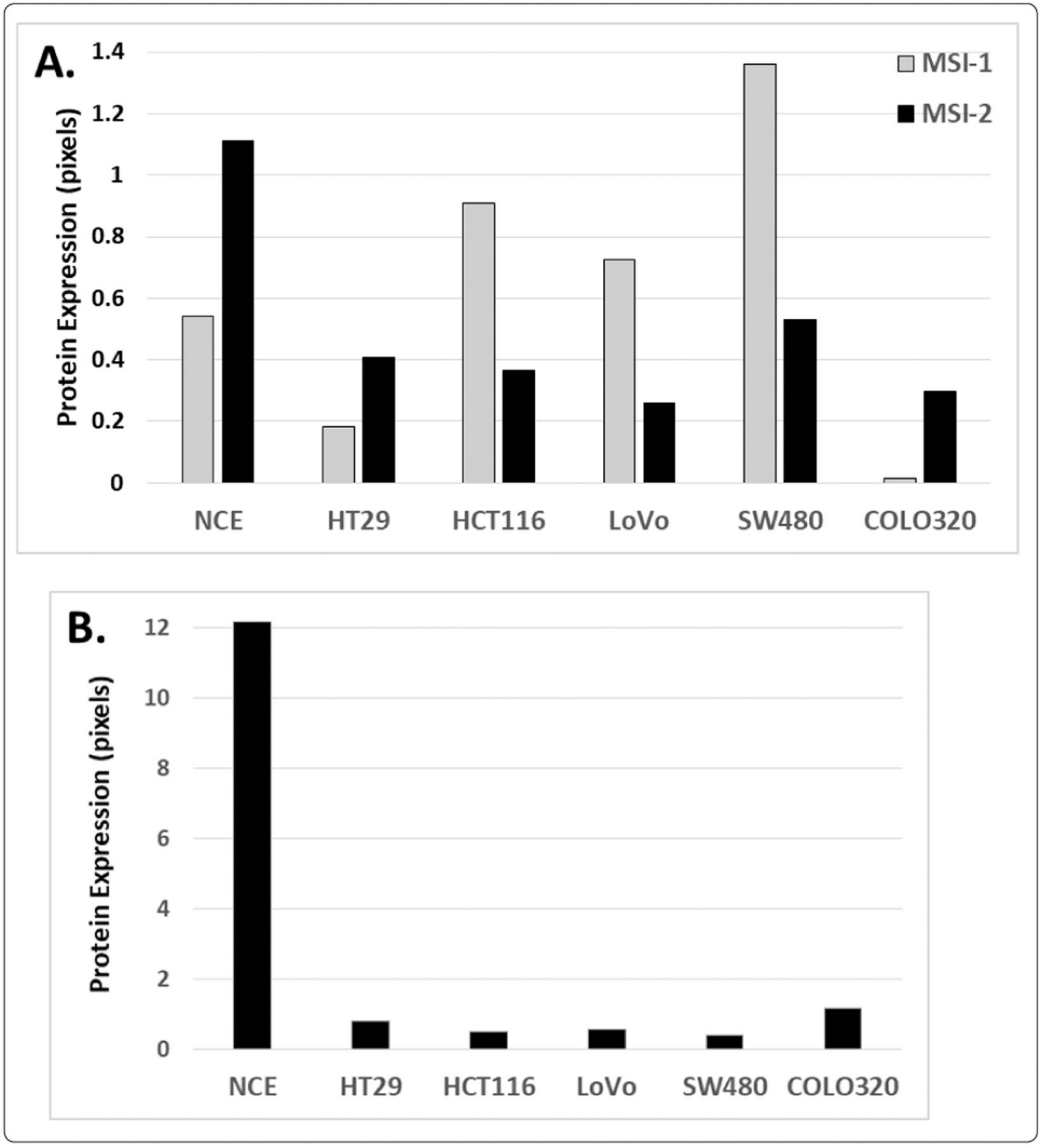
Expression of Musashi and NUMB in CRC cell lines. (A) Expression of MSI-2 and MSI-1 proteins in CRC cells and in normal colonic epithelia. Results show MSI-1 protein expression is variable in CRC cell lines compared to normal colon epithelium, while MSI-2 protein expression is down compared to normal colon epithelium. (B). Expression of NUMB protein in CRC cell lines and in normal colonic epithelia. Results show that CRC cells have decreased NUMB protein expression compared to normal colon epithelium. Protein expression was quantified by densitometric analysis of western blots and normalized to beta actin.

To further investigate the relationship between NUMB and Musashi in CRC, we screened several CRC cell lines and normal colonic epithelium. Western blot analysis showed that MSI-2 and MSI-1 protein expression was variable in CRC cell lines relative to normal colonic epithelium. (Figure 2A). In contrast, our results showed that NUMB protein expression is decreased in CRC cell lines compared to normal colonic epithelium (Figure 2B). This low level of NUMB protein expression in CRC cells is similar to the reduced expression of NUMB we observed in primary CRC tissues. The variability of MSI-1 and MSI-2 protein expression in CRC cell lines may be due to the degree of cellular differentiation of the CRC cells. Indeed, we have previously characterized these cell lines and found significant variability in their immature/stem cell properties and in cellular differentiation [26]. For example, HT29 cells, which have lower levels of MSI-1 and MSI-2, were originally derived from a low grade, well-differentiated tumor and we previously found that HT29 cells have low sternness and high cellular differentiation properties. In contrast, SW480 cells, which have higher levels of MSI-1 and MSI-2, were originally derived from a high grade undifferentiated tumor, and we previously found that SW480 cells have high sternness and low cellular differentiation properties [26,27].

Other investigators have used these CRC cell lines to characterize the role of Musashi/Notch signaling in maintaining sternness. For example, two studies reported that knockdown of MSI-1 in CRC cell lines suppresses cell proliferation, colony formation, tumorsphere formation, and xenograft growth, demonstrating that MSI-1 signaling is important in maintaining sternness and promoting growth of CRC. In a recent study, treatment with Notch-1 siRNA or DAPT Notch-1 antagonist significantly inhibited cell growth, arrested the cell cycle at G1 phase and promoted apoptosis [19,28,29]. While these studies on CRC cell lines establish the importance of MSI-1 and Notch signaling mechanisms in colon tumorigenesis, the role of MSI-2 has been less characterized in human CRC cells.

### Immunostaining for MSI-1, MSI-2 expression in normal and adenomatous colonic crypts

**Figure 3:**
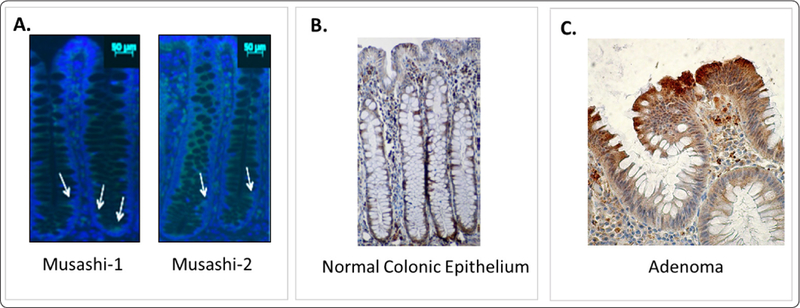
MSI-1 and MSI-2 expressing cells reside in the normal colonic SC niche and are overpopulated in colonic adenomas. (A) Immunofluorescence microscopy of normal human colon epithelium for MSI-1 and MSI-2 reveals positively-staining cells are located in the base of normal colonic crypts. (B) IHC staining with an antibody that reacts to both MSI-1 & MSI-2 shows Musashi-positive cells are mainly in the normal crypt bottom. (C) IHC staining of adenomatous colonic epithelium shows that the Musashi-positive cell population expands in size and shifts toward the crypt top

Here we used two independent staining methods to determine location of MSI-1 and MSI-2 in normal colonic epithelium. As shown in Figure 3A immunofluorescent (IF) staining, MSI-1+ and MSI-2+ cells are located at the bottom of human colonic crypts and mostly reside within the SC niche (levels 1–20). IHC staining of normal colonic epithelium (Figure 3B) with an antibody that reacts to both MSI-1 and MSI-2 shows a similar staining pattern. Other investigators have shown that MSI-1 marks SCs in human colon and mouse intestine [5,6,30]. While Wang, et al. reported that MSI-2 is a marker for murine intestinal SCs, little information was previously known about MSI-2 as a specific marker for human colonic SCs [7]. To date, there has been one study done by Zong, et al. that shows the correlation of MSI-2 overexpression with an unfavorable prognosis and as a potential biomarker of liver metastasis in CRC patients [31]. This study is in agreement with our finding of MSI-2 overexpression in cancerous tissues samples from CRC patients.

In contrast to normal colonic epithelium, IHC staining of adenomatous colonic epithelium shows a different Musashi expression pattern. Staining of colonic adenomas reveals that the MSI-1+ and MSI-2+ population expands in size and shifts in distribution toward the crypt top (Figure 3C). These results concur with our previous findings using immunostaining for the SC marker ALDH1 whereby ALDH1+ cells are located at the normal crypt bottom mostly residing within the SC niche and in adenomatous crypts the ALDH1+ cell population size expands and shifts to the crypt top [14]. In that study, we found similar results for two other SC markers (CD44 and CD133), but ALDH1 staining was more specific for SCs. Overall, these results indicate that during CRC development colonic SCs become overpopulated which contributes to tumor growth.

### The Effect of ATRA-induced differentiation on MSI-2 and NUMB expression in CRC Cells

To further elucidate whether there is a correlation between MSI-2 and NUMB expression during cell differentiation, SW480 and HT29 cells were treated with ATRA. Previously, ATRA treatment has been used to induce differentiation when targeting stem-like cells from gliomas, breast, and colon [32–34]. ATRA treatment has also been shown to downregulate the expression of SC-related genes [35].

**Figure 4:**
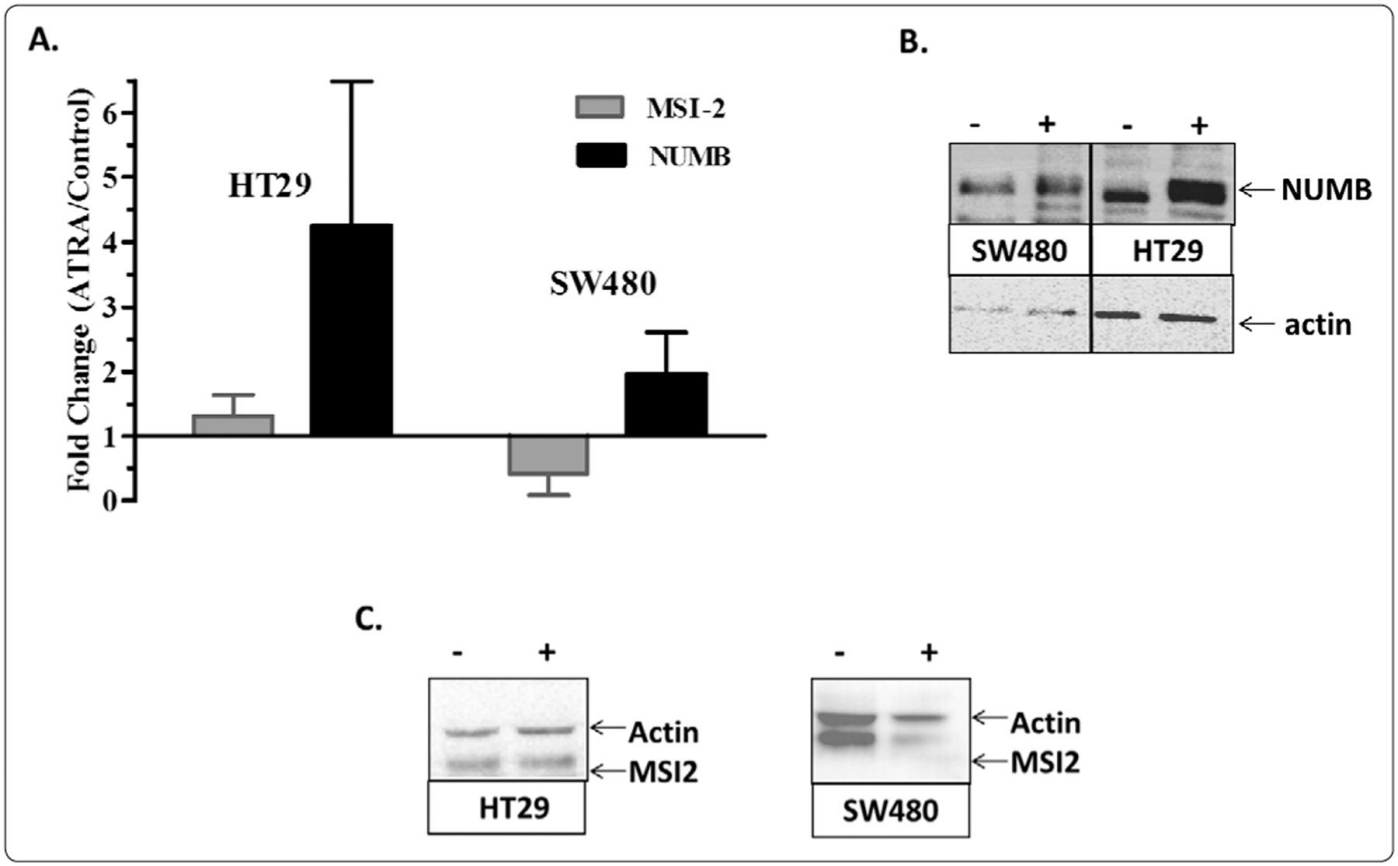
ATRA treatment of CRC cell lines reverts abnormal MSI-2 and NUMB protein levels toward normal epithelial cell protein levels. (A) ATRA- treated cells had no significant change in MSI-2 levels in HT29 cells, but reduced levels of MSI-2 in SW480 cell. Cells treated with ATRA increased the protein expression of NUMB in both cell lines. Cell treatment experiments were performed three times and error bars represent ± SEM. (B and C) Representative western blots of control and ATRA treated HT29 and SW480 cells are shown for MSI-2 and NUMB protein expression, as well as each corresponding beta actin. The lanes are labeled as without (−) or with (+) ATRA.

Our results (Figure 4) show that ATRA treatment did not significantly alter MSI-2 levels in HT29 cells, but reduced levels of MSI-2 in SW480 cells. On the other hand, CRC cells treated with ATRA increased the protein expression of NUMB in both cell lines. These data suggest that MSI-2/NUMB signaling is linked to cellular differentiation status. In this view, during colon tumorigenesis cells become more immature and less differentiated as reflected by overpopulation of MSI-2+ SCs occurring in association with decreased NUMB expression. Upon inducing cellular differentiation with ATRA, the CRC cells decrease MSI-2 expression, and increase NUMB expression, which is a phenotype collectively observed in normal colonic epithelium. In agreement with our findings, it has recently been published that the treatment of gastric cancer SCs with ATRA also upregulates the expression of differentiation markers and reduces the expression of SC markers [36].

## Conclusion

In conclusion, we have demonstrated a link between MSI-2 and NUMB expression in the normal colon and how it becomes disrupted during colon tumorigenesis. This finding is important because previously it was not known if the MSI-2/NUMB pathway contributes to CRC development in humans. Previous studies on mice showed that MSI-2 overexpression induces intestinal tumorigenesis but the mechanism is independent of NUMB [7]. Our findings suggest that, in humans, MSI-2/NUMB signaling is closely associated with colonic SCs, which help maintain normal crypt homeostasis. Moreover, ATRA-induced differentiation is able to restore a normal epithelial cell phenotype with high NUMB and low MSI-2 expression. With the identification of key Musashi/NUMB pathway proteins in normal colonic SCs, how they are aberrantly expressed in CRC cells and how they respond to ATRA treatment provides insight into the design of targeted anti-SC therapies that are more effective and beneficial to patients.

## References

[1] Boman BM, Fields JZ, Bonham-Carter O, Runquist OA (2001) Computer modeling implicates stem cell overproduction in colon cancer initiation, Cancer Res 61: 8408–8411. 11731419

[2] Boman BM, Fields JZ, Cavanaugh KL, Guetter A, Runquist OA (2008) How dysregulated colonic crypt dynamics cause stem cell overpopulation and initiate colon cancer, Cancer Res 68: 3304–3313. 18451157 10.1158/0008-5472.CAN-07-2061

[3] Boman BM, Wicha MS, Fields JZ, Runquist OA (2007) Symmetric division of cancer stem cells--a key mechanism in tumor growth that should be targeted in future therapeutic approaches, Clin Pharmacol Ther 81: 893–898. 17460605 10.1038/sj.clpt.6100202

[4] Knoblich JA (2001) Asymmetric cell division during animal development, Nat Rev Mol Cell Biol 2: 11–20. 11413461 10.1038/35048085

[5] Nishimura S, Wakabayashi N, Toyoda K, Kashima K, Mitsufuji S (2003) Expression of Musashi-1 in human normal colon crypt cells: a possible stem cell marker of human colon epithelium, Dig Dis Sci 48: 1523–1529. 12924647 10.1023/a:1024763723240

[6] Potten CS, Booth C, Tudor GL, Booth D, Brady G (2003) Identification of a putative intestinal stem cell and early lineage marker; musashi-1, Differentiation 71: 28–41. 12558601 10.1046/j.1432-0436.2003.700603.x

[7] Wang S, Li N, Yousefi M, Nakauka-Ddamba A, Li F, (2015) Transformation of the intestinal epithelium by the MSI2 RNA-binding protein, Nat Commun 6: 6517. 25774828 10.1038/ncomms7517 PMC4643281

[8] Okano H, Imai T, Okabe M (2002) Musashi: a translational regulator of cell fate, J Cell Sei 115: 1355–1359. 10.1242/jcs.115.7.1355 11896183

[9] Kudinov AE, Karanicolas J, Golemis EA, Boumber Y (2017) Musashi RNA-Binding Proteins as Cancer Drivers and Novel Therapeutic Targets, Clin Cancer Res 23: 2143–2153. 28143872 10.1158/1078-0432.CCR-16-2728 PMC5413399

[10] Fox RG, Park FD, Koechlein CS, Kritzik M, Reya T (2015) Musashi signaling in stem cells and cancer, Annu Rev Cell Dev Biol 31: 249–267. 26566113 10.1146/annurev-cellbio-100814-125446

[11] Li N, Yousefi M, Nakauka-Ddamba A, Li F, Vandivier L, (2015) The Msi Family of RNA-Binding Proteins Function Redundantly as Intestinal Oncoproteins, Cell Rep 13: 2440–2455. 26673327 10.1016/j.celrep.2015.11.022 PMC4894540

[12] Pasto A, Serafin V, Pilotto G, Lago C, Bellio C, (2014) NOTCH3 signaling regulates MUSASHI-1 expression in metastatic colorectal cancer cells, Cancer Res 74: 2106–2118. 24525742 10.1158/0008-5472.CAN-13-2022

[13] Schulenburg A, Cech P, Herbacek I, Marian B, Wrba F, (2007) CD44-positive colorectal adenoma cells express the potential stem cell markers musashi antigen (msil) and ephrin B2 receptor (EphB2), J Pathol 213: 152–160. 17708598 10.1002/path.2220

[14] Huang EH, Hynes MJ, Zhang T, Ginestier C, Dontu G, (2009) Aldehyde dehydrogenase 1 is a marker for normal and malignant human colonic stem cells (SC) and tracks SC overpopulation during colon tumorigenesis, Cancer Res 69: 3382–3389. 19336570 10.1158/0008-5472.CAN-08-4418 PMC2789401

[15] VanDussen KL, Carulli AJ, Keeley TM, Patel SR, Puthoff BJ, (2012) Notch signaling modulates proliferation and differentiation of intestinal crypt base columnar stem cells, Development 139: 488–497. 22190634 10.1242/dev.070763 PMC3252352

[16] Chiou GY, Yang TW, Huang CC, Tang CY, Yen JY, (2017) Musashi-1 promotes a cancer stem cell lineage and chemoresistance in colorectal cancer cells, Sci Rep 7: 2172. 28526879 10.1038/s41598-017-02057-9 PMC5438397

[17] Bhatlekar S, Viswanathan V, Fields JZ, Boman BM (2018) Overexpression of HOXA4 and HOXA9 genes promotes self-renewal and contributes to colon cancer stem cell overpopulation, J Cell Physiol 233: 727–735. 28464221 10.1002/jcp.25981

[18] Fan LF, Dong WG, Jiang CQ, Xia D, Liao F, (2010) Expression of putative stem cell genes Musashi-1 and betal-integrin in human colorectal adenomas and adenocarcinomas, Int J Colorectal Dis 25: 17–23. 19714342 10.1007/s00384-009-0791-2

[19] Gao C, Han C, Yu Q, Guan Y, Li N, (2015) Downregulation of Msil suppresses the growth of human colon cancer by targeting p21cipl, Int J Oncol 46: 732–740. 25394506 10.3892/ijo.2014.2749

[20] Imai T, Tokunaga A, Yoshida T, Hashimoto M, Mikoshiba K, (2001) The neural RNA-binding protein Musashi1 translationally regulates mammalian numb gene expression by interacting with its mRNA, Mol Cell Biol 21: 3888–3900. 11359897 10.1128/MCB.21.12.3888-3900.2001 PMC87052

[21] Takahashi T, Suzuki H, Imai T, Shibata S, Tabuchi Y, (2013) Musashi-1 post-transcriptionally enhances phosphotyrosine-binding domain-containing m-Numb protein expression in regenerating gastric mucosa, PLoS One 8: e53540. 23308249 10.1371/journal.pone.0053540 PMC3537613

[22] Narayanan P (2015) Musashi-2 promotes c-MYC expression through IRES-dependent translation and self-renewal ability in hepatocellular carcinoma Molecular Microbiology and Immunology, University of Southern California, USC Digital Library.

[23] Sikora K, Chan S, Evan G, Gabra H, Markham N, Stewart J, Watson J (1987) c-myc oncogene expression in colorectal cancer, Cancer 59: 1289–1295. 3545431 10.1002/1097-0142(19870401)59:7<1289::aid-cncr2820590710>3.0.co;2-o

[24] Stewart J, Evan G, Watson J, Sikora K (1986) Detection of the c-myc oncogene product in colonic polyps and carcinomas, Br J Cancer 53: 1–6. 3511934 10.1038/bjc.1986.1 PMC2001472

[25] Konsavage WM, Yochum GS (2014) The myc 3’ wnt-responsive element suppresses colonic tumorigenesis, Mol Cell Biol 34: 1659–1669. 24567369 10.1128/MCB.00969-13 PMC3993608

[26] Modarai SR, Opdenaker LM, Viswanathan V, Fields JZ, Boman BM (2016) Somatostatin signaling via SSTR1 contributes to the quiescence of colon cancer stem cells, BMC Cancer 16: 941. 27927191 10.1186/s12885-016-2969-7 PMC5142402

[27] Opdenaker LM, Modarai SR, Boman BM (2015) The Proportion of ALDEFLUOR-Positive Cancer Stem Cells Changes with Cell Culture Density Due to the Expression of Different ALDH Isoforms, Cancer Stud Mol Med 2: 87–95. 28280782 10.17140/CSMMOJ-2-113 PMC5340268

[28] Sureban SM, May R, George RJ, Dieckgraefe BK, McLeod HL, (2008) Knockdown of RNA binding protein musashi-1 leads to tumor regression in vivo, Gastroenterology 134: 1448–1458. 18471519 10.1053/j.gastro.2008.02.057

[29] Liao W, Li G, You Y, Wan H, Wu Q, (2018) Antitumor activity of Notch 1 inhibition in human colorectal carcinoma cells, Oncol Rep 39: 1063–1071. 29286145 10.3892/or.2017.6176 PMC5802031

[30] Montgomery RK, Breault DT (2008) Small intestinal stem cell markers, J Anat 213: 52–58. 18638070 10.1111/j.1469-7580.2008.00925.x PMC2475558

[31] Zong Z, Zhou T, Rao L, Jiang Z, Li Y, (2016) Musashi2 as a novel predictive biomarker for liver metastasis and poor prognosis in colorectal cancer, Cancer Med 5: 623–630. 26775684 10.1002/cam4.624 PMC4831280

[32] Campos B, Wan F, Farhadi M, Ernst A, Zeppemick F, (2010) Differentiation therapy exerts antitumor effects on stem-like glioma cells, Clin Cancer Res 16: 2715–2728. 20442299 10.1158/1078-0432.CCR-09-1800

[33] Zanetti A, Affatato R, Centritto F, Fratelli M, Kurosaki M, (2015) All-trans-retinoic Acid Modulates the Plasticity and Inhibits the Motility of Breast Cancer Cells: ROLE OF NOTCH 1 AND TRANSFORMING GROWTH FACTOR (TGFbeta), J Biol Chem 290: 17690–17709. 26018078 10.1074/jbc.M115.638510 PMC4505019

[34] Chanchevalap S, Nandan MO, Merlin D, Yang VW (2004) All-trans retinoic acid inhibits proliferation of intestinal epithelial cells by inhibiting expression of the gene encoding Kruppel-like factor 5, FEBS Lett 578: 99–105. 15581624 10.1016/j.febslet.2004.10.079 PMC1599793

[35] Pellegrini M, Filipponi D, Gori M, Barrios F, Lolicato P (2008) ATRA and KL promote differentiation toward the meiotic program of male germ cells, Cell Cycle 7: 3878–3888. 19098446 10.4161/cc.7.24.7262

[36] Nguyen PH, Giraud J, Staedel C, Chambonnier L, Dubus P, (2016) All-trans retinoic acid targets gastric cancer stem cells and inhibits patient-derived gastric carcinoma tumor growth, Oncogene 35: 5619–5628. 27157616 10.1038/onc.2016.87

